# A Low Percent Ethanol Method for Immobilizing Planarians

**DOI:** 10.1371/journal.pone.0015310

**Published:** 2010-12-14

**Authors:** Claire G. Stevenson, Wendy Scott Beane

**Affiliations:** Biology Department, Tufts Center for Regenerative and Developmental Biology, Tufts University, Medford, Massachusetts, United States of America; King's College London, United Kingdom

## Abstract

Planarians have recently become a popular model system for the study of adult stem cells, regeneration and polarity. The system is attractive for both undergraduate and graduate research labs, since planarian colonies are low cost and easy to maintain. Also *in situ* hybridization, immunofluorescence and RNA-interference (RNAi) gene knockdown techniques have been developed for planarian studies. However, imaging of live worms (particularly at high magnifications) is difficult because animals are strongly photophobic; they quickly move away from light sources and out of frame. The current methods available to inhibit movement in planarians include RNAi injection and exposure to cold temperatures. The former is labor and time intensive, while the latter precludes the use of many fluorescent reporter dyes. Here, we report a simple, inexpensive and reversible method to immobilize planarians for live imaging. Our data show that a short 1 hour treatment with 3% ethanol (EtOH) is sufficient to inhibit both the fine and gross movements of *Schmidtea mediterranea* planarians, of the typical size used (4–6 mm), with full recovery of movement within 3–4 hours. Importantly, EtOH treatment did not interfere with regeneration, even after repeated exposure, nor lyse epithelial cells (as assayed by H&E staining). We demonstrate that a short exposure to a low concentration of EtOH is a quick and effective method of immobilizing planarians, one that is easily adaptable to planarians of all sizes and will increase the accessibility of live imaging assays to planarian researchers.

## Introduction

As a model system, planarians are commonly used to study stem cell-dependent regeneration because of their extensive regenerative abilities and large population of stem cells that comprise roughly 30% of adult tissues [1], [2]. Planarians are non-parasitic, soft-bodied flatworms with a central nervous system consisting of a bi-lobed cephalic ganglia (brain) and two ventral nerve chords extending to the posterior [3], [4]. An extensible pharynx on the ventral side is used as both a mouth and anus and is connected to a combined gastrovascular digestive tract [2]. During regeneration a burst of mitotic activity produces a mass of new, unpigmented tissue at the wound site (blastema) from which, combined with remodeling of existing tissues (morphallaxis), the worm can replace any and all portions of its body [1].

**Figure 1:**
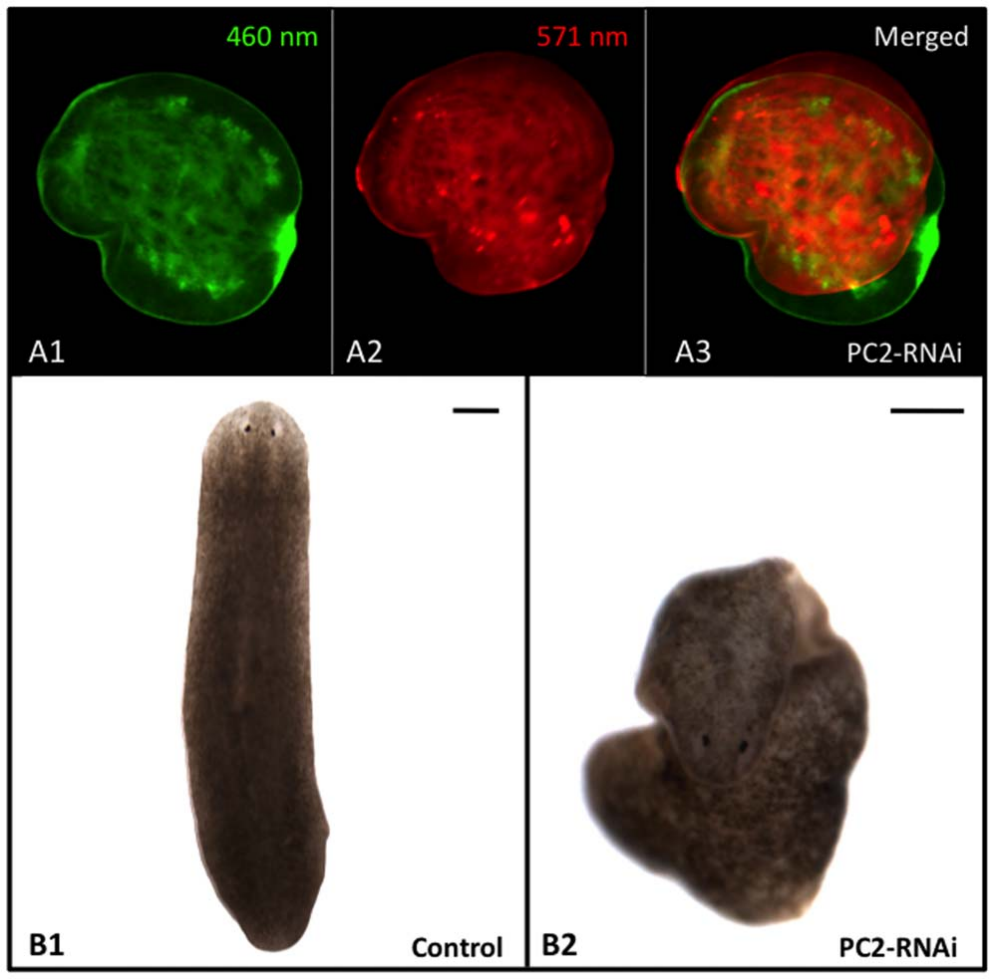
Need for An Improved Planarian Immobilization Technique. (**A**) *Membrane Voltage Reporter Dye Assay*. When consecutive images of a single planarian trunk fragment at 24 hours of regeneration showing (**A1**) CC2 (460 nm) and (**A2**) DiBAC (517 nm) staining are (**A3**) overlaid, the failure of PC2-RNAi injection to prevent fine movements is illustrated. This assay requires correction of CC2 fluorescence using DiBAC, which is not possible when overlaid images are not in register (as in A3). Regenerates had to be injected with PC2-RNAi 2 weeks prior to amputation, well in advance of imaging. Anterior is to the right. (**B**) *Characteristic Morphology*. (B1) Untreated control and (B2) PC2-RNAi-injected worm (displaying typical curled phenotype). Anterior is up. Scale bars = 250 µm.

Research involving the live imaging of planarians has been limited, largely because flatworms are photophobic; when placed under a microscope, worms move quickly out of the imaging field to avoid the light. This is particularly problematic for dual-reporter assays where images from different spectra must be overlaid (for example see Figure 1A). These assays require imaging at high magnifications (where even small movements can create issues), and they are particularly common for the visualization of biophysical processes such as membrane voltage and pH gradients [5]. Recent investigations into the regulation of stem cell proliferation/maintenance and regeneration in planarians has highlighted the importance of biophysical mechanisms during these processes [6], [7], [8], [9], [10], necessitating better methods to inhibit movements in live worms.

No means to paralyze planaria have been reported, and very few methods have even been shown to reduce movements in live animals (immobilization). Although no standard method to immobilize planarians exists, the two most successful methods currently reported in the literature are: exposure to cold temperatures with chilled water, ice, or a cold plate [11], [12]; and RNA interference (RNAi) against Pro-hormone convertase 2 (PC2) [5], [9]. (PC2, a neuropeptide processor, is expressed throughout the planarian central nervous system [13].) Unfortunately, cold is not a viable option for some imaging assays, like the membrane voltage assay shown in Figure 1, because many of the fluorescent reporter dyes are temperature sensitive. The alternative, injection with PC2-RNAi, is an improvement over cold but still not ideal. While PC2-RNAi-injected worms do lose gross movements (such as moving across a Petri dish), they continue to exhibit some fine movements (such as rippling, contraction or expansion of the body while stationary), which prevents multiple images from a single worm being easily overlaid in register (Figure 1A3). PC2 inhibition also causes worms to curl up and resist lying flat (Figure 1B), which can make imaging live worms challenging. An improved method for immobilizing planarians is needed.

Planarians move via both ventral ciliated epithelial cells used for gliding, and muscles which are used to negotiate obstacles [14], [15]. Although the exact mechanism is unknown, it is thought that PC2 inhibition effects only muscular movement. A treatment that inhibits the movement of the ventral cilia (alone or combined with the musculature) may be more effective for immobilizing planarians. It has been reported that 1% ethanol (EtOH) removes most epithelial cilia from the planaria *Dugesia japonica*; and although movement was not investigated in depth, it was noted that 1% EtOH did not paralyze worms [14]. In this study, we aimed to carefully investigate the relationship between EtOH treatment and inhibition of movement in flatworms, towards the establishment of an improved technique to immobilize live planarians. Our data revealed that brief exposure to a low percent EtOH (3%) inhibits planarian movement without adverse toxicity.

## Results

### A Brief 3% Ethanol Exposure Inhibits Planarian Movement

For these experiments we used the planaria *Schmidtea mediterranea*, a species with a sequenced genome that is widely used in the planarian community, particularly for molecular analyses and reverse genetics. Preliminary studies were conducted with a number of different compounds predicted to inhibit planarian mobility (data not shown). The compounds selected were roughly divided into two groups: those targeting muscle movements (haloperidol, reserpine and sulperide), and those aimed at removing cilia (a triton-based extracting solution, chloral hydrate, and ethanol). Of these, a 1 hour treatment with 3% ethanol (EtOH) was found to be the most effective at immobilizing planarians 4–6 mm in length (a standard size used in planarian assays) without toxicity.

In this study, exposure (treatment length) was defined as a period of incubation in 3% EtOH (usually 1 hour, unless otherwise stated) followed by washing out of EtOH. Thus, treated (immobilized) planarians are worms that have been exposed to EtOH and then rinsed in plain worm water. Treated worms were always scored and used after removal from EtOH, because worms that are still in low percent EtOH solutions are prone to twitching movements. Therefore, full immobilization was best seen only after washing out of EtOH. Recovery from EtOH immobilization was defined as treated worms which had regained movements.

**Figure 2:**
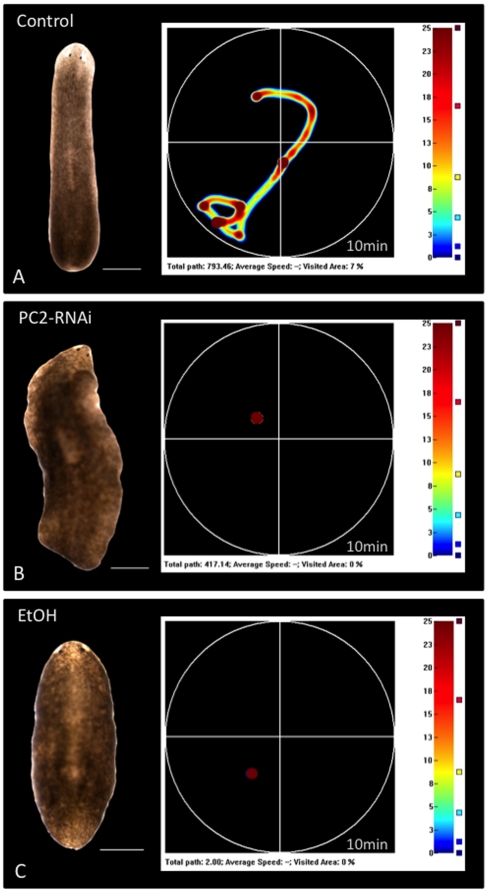
A 3% Ethanol Treatment Inhibits Gross Movements. *Behavioral Analysis of Gross Movements (scored by distance traveled across a Petri dish)*. Computer vision system tracks worm movement over time (10 minutes). Heat maps (curiosity plots) graph areas not visited (black), least time visited (blue), and most time visited (red) for each trial. To the left is displayed the specific worm that generated each map. (**A**) Untreated control, (**B**) PC2-RNAi injected and (**C**) EtOH-treated (for 1 hour) worms. Note that although both PC2 inhibition and EtOH treatment block gross movements (0% of dish explored for each), the morphology of EtOH-treated worms is better. Anterior is up. Scale bars = 500 µm.

The gross morphology of EtOH-treated worms was observed to differ from untreated worms (appearing “scrunched” with head and tail pulled close to the body); however this change in morphology was less severe than that seen with PC2-RNAi-injected worms (Figure 2). PC2-RNAi injection is one method currently available to immobilize planarians that is suitable for use with electrophysiology and fluorescent reporter dye assays. However, it results in worms that have uneven margins (ruffled edges) and that are prone to curl in on themselves rather than lie flat (Figure 1B and 2B). For instance, the worm in Figure 2B was straightened with tweezers prior to photographing, although its tail resisted straightening and remained curled underneath the worm. In contrast, while EtOH-treated worms appeared “scrunched,” they remained flat with no ruffling or curling and without the need to manipulate individual worms prior to taking photos (Figure 2C).

To assay for immobilization, both gross and fine movements were examined. Gross movements, defined by distance traveled across a surface such as a Petri dish, were quantified using a machine that measures the location (x,y coordinates) of a worm over time [19]. Trials were run for 10 minutes in the dark, with coordinates recorded at 5 hertz (200 ms intervals). Representative heat maps, or curiosity plots, of movement during a single trial are shown in Figure 2, with the corresponding worm pictured beside the map it generated. When placed in a new environment, planarians typically undergo a brief period of exploration; on average, untreated control planarians explored 10.5% of the dish during a trial (n = 12). However, neither PC2-RNAi-injected nor EtOH-treated worms exhibited any exploratory behavior at all; every planarian tested for both treatments remained stationary (0% explored, PC2-RNAi n = 6, EtOH-treated n = 7) wherever they settled after being placed in the dish (for both p<0.005). The data demonstrate that PC2 inhibition and EtOH immobilization are equally effective at blocking gross movements.

**Figure 3:**
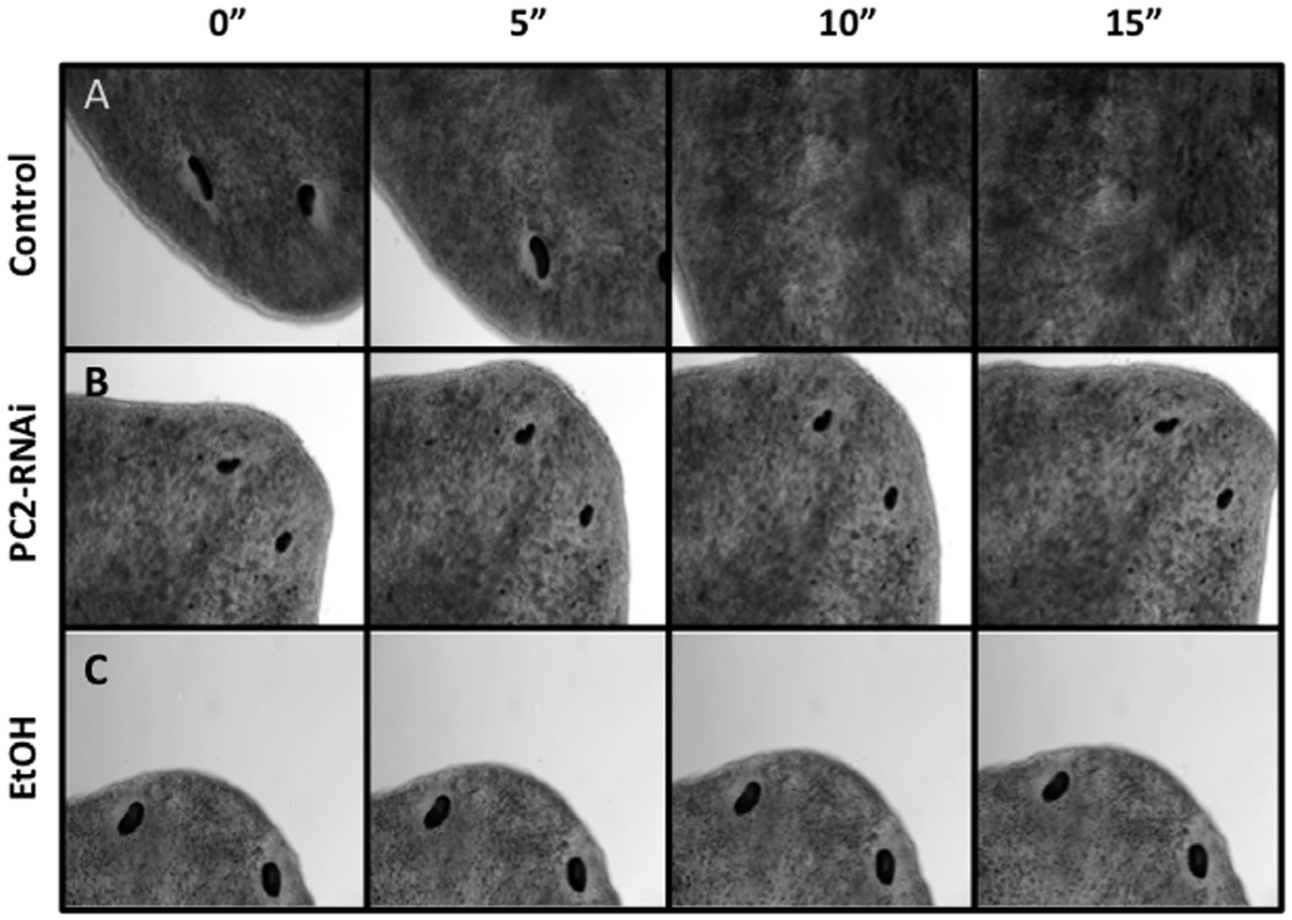
A 3% Ethanol Treatment Inhibits Fine Movements. *Analysis of Fine Movements (body movements while stationary)*. Time lapse images of worms taken over 15 seconds, highlighting the need for immobilization of live planarians when imaging. (**A**) Untreated control worm moved its head completely out of frame in less than 10 seconds. (**B**) A PC2-RNAi injected worm (without gross movements) remained in frame; however fine movements were not inhibited, as seen by its head progressively extending across the frame. (**C**) An EtOH-treated worm stayed completely in frame without any fine movements. (The assay required loose mounting, resulting fluid drift, but note that the eye/body positions remain the same).

Fine movements (movements of the body while staying in one place) were assayed by high magnification. Worms were lightly placed between a slide and cover slip (to avoid damaging animals and allow for free movement), and time lapse images were taken of a worm's head at 20× over 15 seconds (Figure 3). The head region was selected because even worms with no gross movements tend to extend or retract their heads, and/or rock them from side-to-side, when exposed to intense light (such as under a microscope). The images of an untreated worm show why immobilization of live worms is important: within 10 seconds a control planaria had moved its head completely out of frame (Figure 3A). PC2-RNAi-injected planarians are easier to image because they remain mostly in frame as a result of gross movement inhibition. However PC2-RNAi injection fails to inhibit fine movements, as seen by a PC2-inhibited worm's ability to extend its head completely across the image frame (Figure 3B). In comparison, the head of the EtOH-treated planarian remained in one place throughout the entire time lapse (Figure 3C), without any back and forth movements observed with PC2-RNAi injection. (The slight change in position of the EtOH worm in Figure 3C reflects fluid drift, due to the loose placement between slide and coverslip required to allow free movements. Note that the margins of the animal remain the same.) These data show that unlike PC2-RNAi injection, 3% EtOH exposure inhibits planarian fine movements, suggesting EtOH treatment is an improvement over current techniques.

### Animal Size Affects Treatment Time but Not Recovery Time

**Figure 4:**
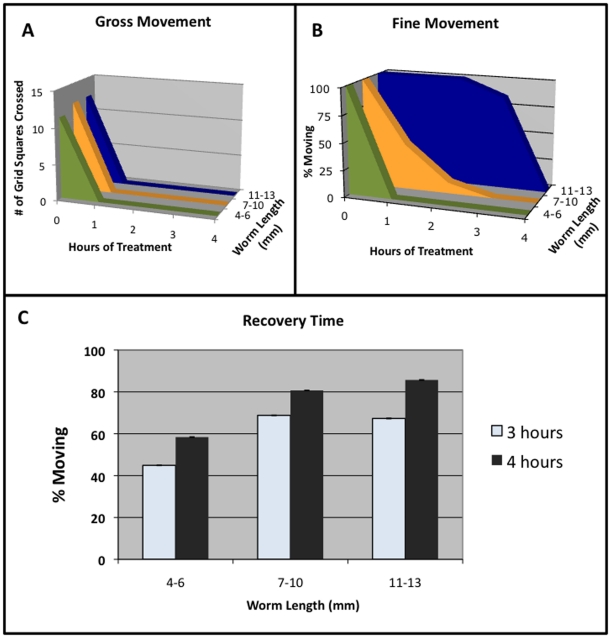
Worm Size Affects Treatment Times for Fine, But Not Gross, Movements. (**A–B**) *Treatment Time Needed to Stop Movement vs. Worm Length*. (**A**) Gross movements are not affected by exposure time, as worms of all lengths are inhibited within 1 hour. 4–6 mm worms (n = 22), 7–10 mm (n = 10), 11–13 mm (n = 37). (**B**) Fine movement inhibition is linked to exposure time, as longer worms require longer treatments before movement is inhibited. 4–6 mm worms (n = 10), 7–10 mm (n = 10), 11–13 mm (n = 12). (**C**) *Recovery time vs. Worm Length*. The majority of worms regain all movements by 3–4 hours after being washed out of EtOH, showing that recovery time is also not affected by worm size. 4–6 mm worms (n = 89), 7–10 mm (n = 114), 11–13 mm (n = 42). Error bars reflect 95% Confidence Intervals.

Our data revealed that a single 1 hour exposure to 3% EtOH was sufficient to inhibit both gross and fine movements in planarians 4–6 mm in length, the most commonly used worm size. However, we also performed analyses of EtOH treatment to investigate its usefulness in larger worms. Our data revealed that fine movement inhibition by EtOH treatment is affected by animal size (Figure 4). For worms of all length, gross movements were inhibited by EtOH treatment within 1 hour (4–6 mm, 100%, n = 168; 7–10 mm, 100%, n = 40; and 11–13 mm, 100%, n = 37; Figure 4A). However, inhibition of fine movements required longer EtOH exposure times for larger worms (Figure 4B). While 4–6 mm long planarians had their fine movements inhibited by EtOH within the same 1 hour that inhibited their gross movements (98.9%, n = 168), it required 3 hours for 7–10 mm worms (100%, n = 136) and 4 hours for 11–13 mm worms (100%, n = 49) to inhibit fine movements. These data show that EtOH treatment can be useful to immobilize planarians of all sizes, given the appropriate exposure time.

Our investigations revealed that while EtOH treatment immobilizes planarians, it does not paralyze them. Immobilized worms still responded to tactile stimulation, such being touched by a pipette, with a temporary burst of fine (but not gross) movement. When moved from one dish to another, EtOH-immobilized worms also responded with a short period of fine movement that ceased within 3–5 minutes for most worms (4–6 mm, 86.7%, n = 168; 7–10 mm, 65.3%, n = 49; and 11–13 mm, 89.7%, n = 39). A 100% penetrance of fine movement cessation could be achieved for all worm sizes by allowing treated worms to remain undisturbed, even under bright light, for up to 10 minutes (or 15 minutes for large worms). The data suggest that EtOH treatment is sufficient to completely disrupt planarian photophobic responses to light, although they are still somewhat able to respond to mechanical stimulation.

An important criteria in searching for a better method of planarian immobilization was that the treatment be reversible. Our data reveal that regardless of size, planarians began to recover movement within 3–4 hours of being washed out of 3% EtOH (4–6 mm, 58.4%, n = 89; 7–10 mm, 80.7%, n = 114; and 11–13 mm, 85.7%, n = 42; Figure 4C). It should be noted that recovery of fine movements was observed to slightly precede gross movement recovery. This 3–4 hour timeframe is sufficient to allow researchers to complete most assays that require immobilization of live worms prior to the onset of recovery.

### Immobilization with 3% Ethanol Does Not Alter Regeneration or Lyse Epithelia

**Figure 5:**
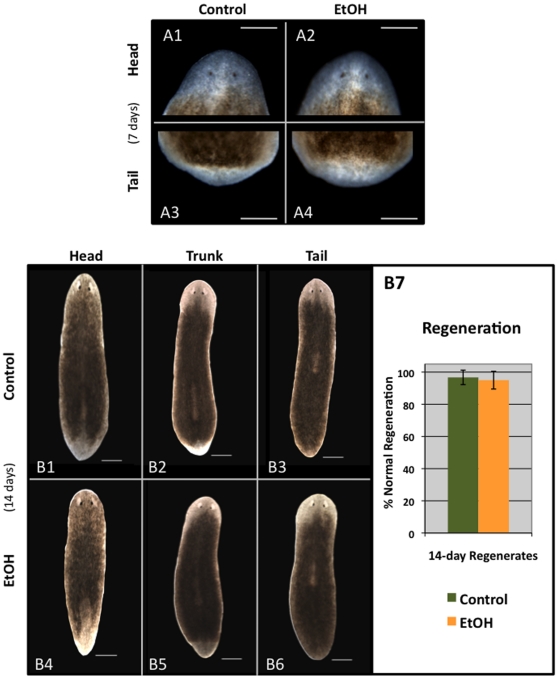
A 3% Ethanol Treatment Does Not Affect Regeneration. *Regeneration Morphology*. Worms were washed out of a 1-hour ETOH treatment and immediately cut into thirds (creating head, trunk and tail fragments). (**A**) Analyses at 7 days of regeneration. No delays in either eye development (A1–A2) or blastema formation (A3–A4) were observed between control (A1, A3) and EtOH-treated (A2, A4) regenerates. Trunk fragments shown. Scale bars = 250 µm. (**B**) Analysis at 14 days of regeneration shows that untreated control (B1–B3) and EtOH-treated (B4–B6) worms equally form blastemas and regenerate with correct anterior/posterior polarity and patterning. Scale bars = 500 µm. (B7) There is no significant difference between control (n = 58) and EtOH-treated (n = 57) regenerates (p = 0.78). Error bars reflect 95% Confidence Intervals. Anterior is up.

The majority of studies using planarians focus on their regenerative abilities. Thus is it vital that any method to inhibit worm movement does not subsequently alter regeneration. To assay for regeneration effects, 4–6 mm planarians were exposed to 3% EtOH for 1 hour, then washed out of EtOH and scored to assure 100% immobilization. Worms were immediately cut into thirds (resulting in head, trunk and tail fragments) and allowed to regenerate for two weeks prior to scoring (Figure 5). We observed no differences or delays in the timing or development of the blastema, pharynx, and/or eyes between control and EtOH-treated regenerates. For instance, at 7 days of regeneration, both control and EtOH-treated trunk fragments had similar eye and blastema formation (Figure 5A). Additionally, we investigated the effects of EtOH exposure on mitotic activity. 4-day regenerates were fixed and stained for phosphorylated histone H3 (H3P) that marks stem cells (neoblasts), the only mitotically-active planarian cells. The data showed no significant difference between the number of neoblasts in control and EtOH-treated regenerates (control = 148.3, n = 10; EtOH = 141.6, n = 10; p = 0.65).

Together, the data revealed that a short 3% EtOH treatment does not affect regeneration. There was no significant difference between untreated and EtOH-treated regenerates at 2 weeks of regeneration (p = 0.78), which both produced blastemas and regenerated with correct anterior/posterior polarity and no eye patterning defects (Figure 5B). To rule out possible effects from repeated exposure to 3% EtOH, planarians were treated for 1 hour and scored to assure immobilization once each day for 4 consecutive days; on the fifth day worms were cut in half (resulting in head and tail fragments); then at 2 weeks regenerates were scored for regeneration. Despite repeated exposures, EtOH treatment did not result in any regeneration defects (100%, n = 68). These data demonstrate that a short 3% EtOH treatment of intact worms does not affect blastema formation or patterning during regeneration.

**Figure 6:**
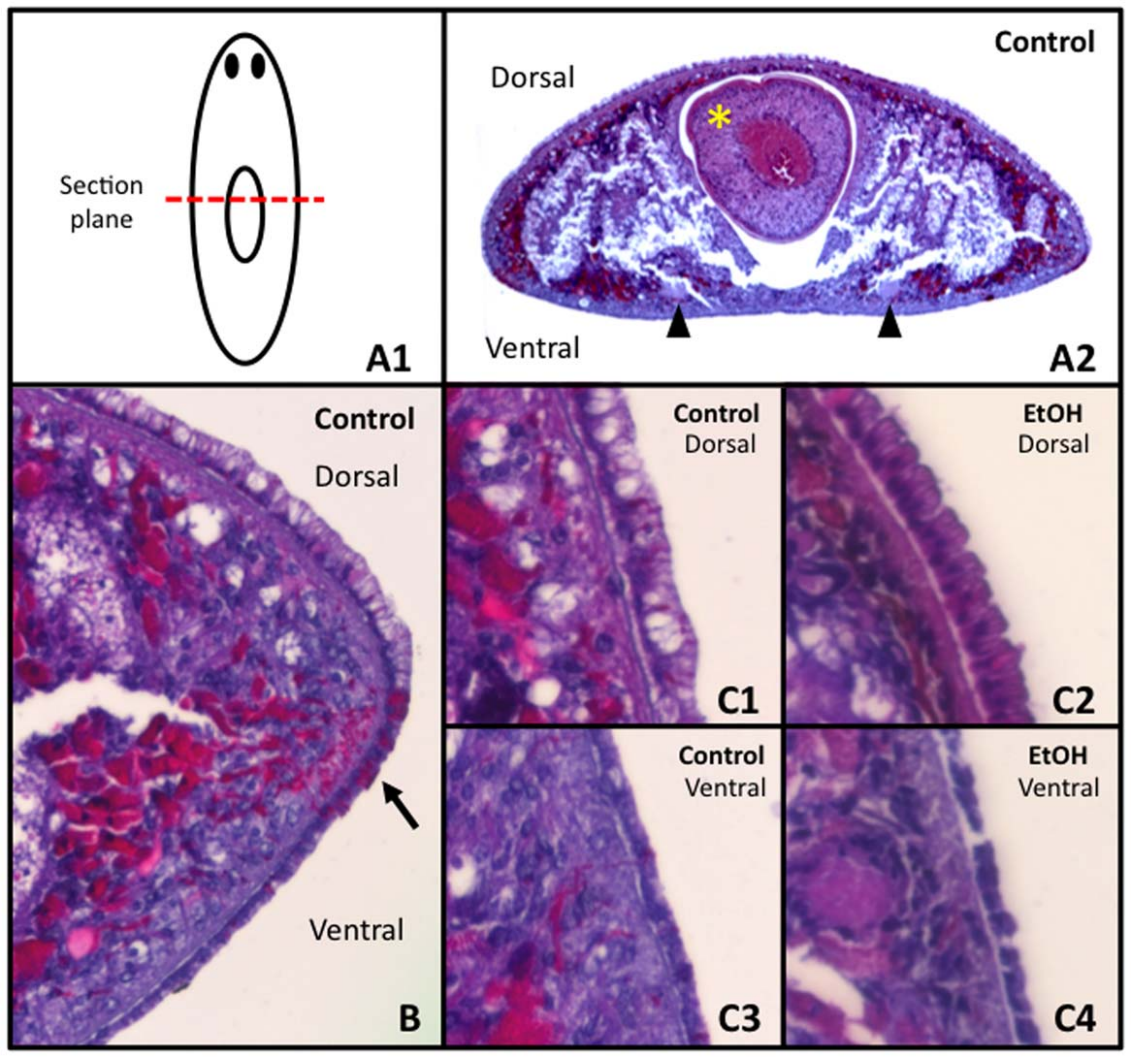
A Short 3% Ethanol Treatment Does Not Cause Epithelial Cell Lysis. *H&E Staining Analysis*. (**A1**) Diagram of sections. (**A2**) Transverse section of control worm. Yellow asterisk  =  pharynx. Black arrowheads  =  round ventral nerve cords (which denote the ventral surface). (**B**) Closer view of an untreated control section, illustrating the larger columnar dorsal epithelial cells, the smaller cuboidal ventral epithelial cells, and the adhesive cells that lie at the dorsal/ventral margin which stain bright pink (arrow). (**C1**) Control dorsal, (**C2**) EtOH-treated dorsal, (**C3**) control ventral and (**C4**) EtOH-treated ventral epithelia showing that EtOH treatment does not disrupt epithelial cell membranes.

Exposure to high percent EtOH (≥70%) is routinely used to sacrifice planarians through disruption of cell membranes (lysis), and this potentially could be an unwanted side effect of EtOH immobilization. To determine whether brief 3% EtOH exposure results in cell lysis, worms were fixed immediately after a 1 hour treatment, then cut into thin, transverse paraffin sections and stained with hematoxylin and eosin (H&E, Figure 6). In these sections the dorsal and ventral sides can be distinguished from each other by the location of the ventral nerve chords (arrowheads in Figure 6A2). A close up of the body margin (Figure 6B) reveals characteristic differences between the single layer of epidermal cells on the dorsal side (wider, columnar cells) and the ventral side (shorter, cuboidal cells). At the margin between dorsal and ventral tissues are the adhesive cells, which can be seen as a layer of bright pink cells extending out from the epidermis (arrow in Figure 6B).

A comparison between untreated control and EtOH-treated sections revealed differences in the contents of some cells within both the epidermis and the parenchyma. Throughout the dorsal epithelial layer in controls, cells with white fluid-filled regions could be observed (Figure 6C1), but these were missing in EtOH-treated sections (Figure 6C2). These unstained areas in the dorsal epithelium are morphologically reminiscent of H&E-labeled mucus-producing cells in human respiratory epithelia [22], [23], suggesting that EtOH treatment may affect mucus production. Similarly, the mesoderm (parenchymal cells) in both dorsal and ventral halves of control sections contained areas abundant in bright pink eosin staining (Figure 6C1, 6C3), which were absent in EtOH-treated tissues (Figure 6C2, 6C4). Importantly, examination of sections showed no evidence of cell lysis in either untreated or EtOH-treated worms, demonstrating that a brief exposure to 3% EtOH does not disrupt epithelial cell membrane integrity in planarians.

### 3% Ethanol Treatment Causes Epidermal Cilia Loss

**Figure 7:**
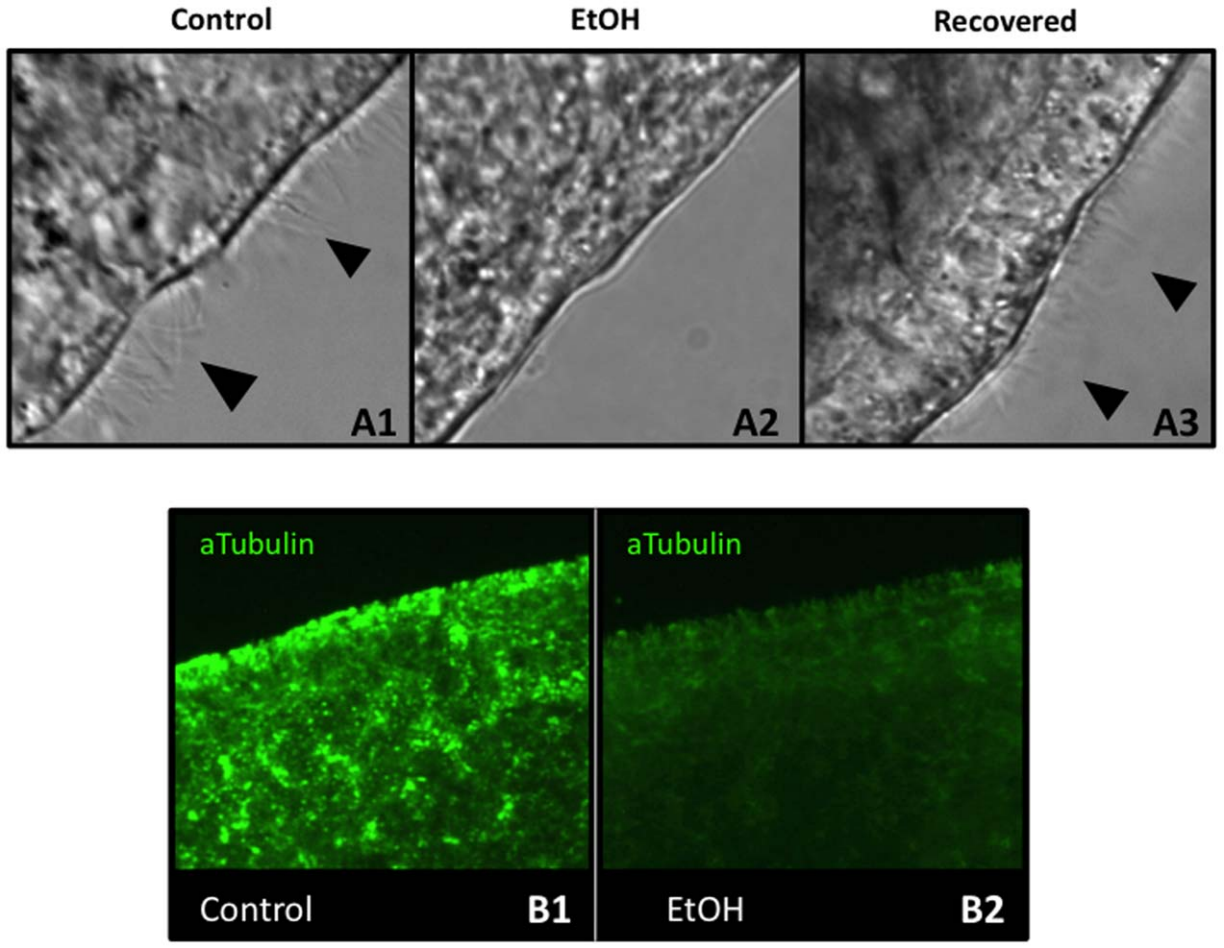
A 3% Ethanol Treatment Removes Epithelial Cila. (**A**) *Analysis of Cilia in the Head Region*. (A1) Untreated control worms are ciliated. (A2) After a 1-hour EtOH treatment, worms have become deciliated. (A3) After 4 hours of recovery, EtOH-immobilized worms are again ciliated. n = 11 for each. Black arrowheads  =  cilia. (**B**) *Anti-acetylated tubulin staining of cilia on the ventral surface*. (B1) Control worms are heavily ciliated (bright green staining). (B2) After a 1-hour EtOH exposure, epidermal ciliary staining is lost. Pre-pharyngeal region is shown.

The mechanism by which 3% EtOH inhibits both the gross and fine movements is not known. It has previously been shown that low concentrations of EtOH can remove the epidermal cilia of planarians [14]. Planarians move in part by ciliary gliding, producing a layer of mucus in which the ventral ciliated cells can propel the worm without using muscles [15], [24]. Therefore, we investigated loss of cilia as one potential mechanism by which a brief 3% EtOH exposure results in loss of planarian movement. Untreated, EtOH-treated (immobilized), and recovered planarians (treated worms that had regained movements) were assayed for the presence or absence of cilia in the heavily-ciliated head region (Figure 7A). Control, untreated worms had clearly visible cilia (Figure 7A1). This dense ciliated layer was no longer observed in EtOH-immobilized worms, with most cilia now lost (Figure 7A2). When EtOH-immobilized worms had recovered (3–4 hours after washing out of EtOH), cilia were once again observed (Figure 7A3). This recovery of cilia is consistent with the timing of gross movement recovery (Figure 4C). These data suggest that EtOH treatment does remove epidermal cilia from planarians, which contributes at least in part to their immobilization.

To corroborate these data, control and EtOH-treated worms were fixed and stained with an acetylated tubulin antibody (Figure 7B), which labels planarian epidermal cilia [25], [26], as well as the ciliated flame cells of the excretory system located beneath the epidermis [27]. Using this method, the dense covering of cilia on the ventral surface in control worms was easily visualized in bright green (Figure 7B1). However, after a 1-hour exposure to EtOH, epidermal ciliary staining was lost, although the dimmer, sub-epidermal staining was still apparent (Figure 7B2). This suggests that EtOH-treatment is sufficient to remove motile cilia from surface cells (without affecting non-epidermal ciliated cell types). The results also demonstrate that EtOH exposure does not interfere with common assays, such as immunohistochemistry, used by planarian researchers. Overall, our data show that treatment with 3% EtOH for 1 hour is an improved, simple method for immobilizing planarians that reversibly inhibits fine movements without adversely affecting regeneration.

## Discussion

These experiments highlight a new method of immobilizing live planarians: a short exposure to low percent ethanol (EtOH). Here we demonstrate that both the fine and gross movements of 4–6 mm *Schmidtea mediterranea* flatworms, the most commonly used size, are inhibited by a 1 hour treatment with 3% EtOH. Preliminaries studies suggest that EtOH treatment similarly immobilizes other planarian species, although the concentration required may need adjustment (for instance, 5% EtOH works well for *Dugesia japonica*, data not shown). This method is an improvement over currently available methods, such as PC2 inhibition and exposure to cold, and is also more compatible with live worm assays using dual-reporter dyes that require superimposing images of two or more fluorophores (frequently as a loading control for dye uptake).

**Figure 8:**
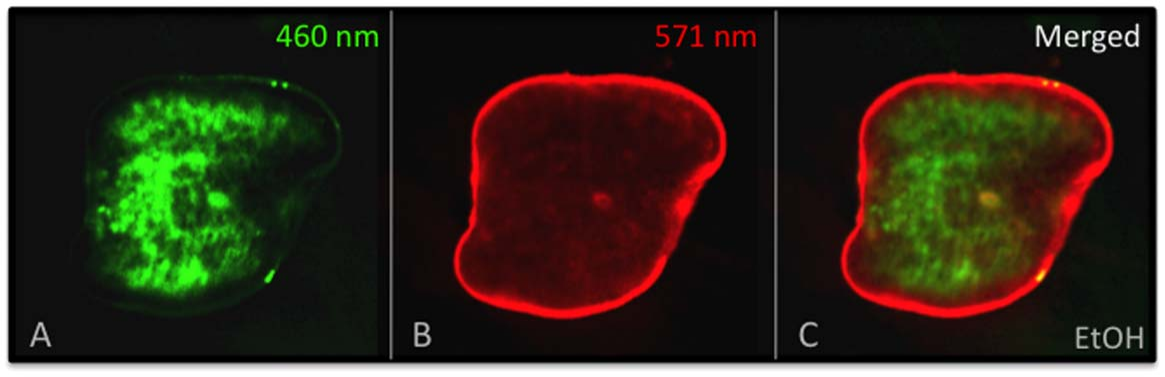
EtOH Immobilization is an Improved Method for Reporter Dye Assays. *Membrane Voltage Reporter Dye Assay*. When consecutive images of a single planarian trunk fragment at 24 hours of regeneration, showing (**A**) CC2 (460 nm) and (**B**) DiBAC (517 nm) staining, are (**C**) overlaid, the EtOH-mediated fine movement inhibition results in images that merge completely in register, allowing for the DiBAC correction of CC2 fluorescence required by the assay. Compare this overlay with the failed overlay using PC2-RNAi in Figure 1A. Regenerates were treated with 3% EtOH just prior to reporter dye incubation and imaging. Anterior is to the right.

For example, compare overlaid, consecutive images of reporter dyes in PC2-RNAi-injected (Figure 1A) and EtOH-treated planarians (Figure 8). PC2-RNAi injection (prior to amputation) failed to inhibit fine movements in regenerating fragments; thus merging signals from two different flourophores results in an overlaid image completely out of register (Figure 1A3). Compare this with EtOH immobilization of regenerating fragments (after amputation), where merging wavelengths results in an overlaid image that remains in register (Figure 8C). Our data showed that there were no adverse effects on either cell membrane integrity or later regeneration (even with repeated exposures), suggesting that EtOH immobilization for live imaging with reporter dyes can be combined with subsequent tracking of worms over time for scoring or re-imaging.

Our data revealed a correlation between worm size and exposure time; larger worms required slightly longer treatment times to fully inhibit all movements (Figure 4B). It should be noted that long-term exposures to 3% EtOH were found to be toxic; for instance, we have found that overnight treatment (>16 hours) of even the largest (11–13 mm) worms induced head regression, lesion formation and occasionally death. This correlates with previous studies reporting that prolonged exposure to even low levels of EtOH (<1%) produced toxic effects and abnormal development, particularly if worms were continuously exposed during regeneration [28]. The correlation between worm size and treatment length was true for fine movements but not for gross movements. Regardless of size, all planarians tested lost gross movements within 1 hour (Figure 4A). Recovery of movement also did not correlate with worm size; 3–4 hours of recovery was sufficient for most worms to recover movement (Figure 4C). Different treatment timescales for different movements suggests that EtOH-mediated inhibition of gross and fine movements may occur by different mechanisms.

Gross movement inhibition by EtOH due to cilia removal is consistent with both our timing results (all worms lost gross movement by 1 hour regardless of size) and cilia analyses showing that after only 1 hour of EtOH treatment epidermal cilia are lost (Figure 7). This is further consistent with our data showing that worms recover gross movement around 3–4 hours, regardless of the length of EtOH exposure. By this time (4 hours after washing out of EtOH), epidermal cilia have reappeared in treated worms (Figure 7A3). This timeframe is sufficient to allow planarians to regrow their cilia. Studies have shown that deciliated sea urchin embryos begin to regenerate epidermal cilia (as a group) well before 2 hours, and by 4 hours their cilia have reached 70% of their original length [29], [30]. EtOH-meditated cilia removal most likely occurs by scission, the mechanism by which deciliation of sea urchin embryos and paramecium occurs [31], [32], [33], perhaps caused by the influx of calcium into the plasma membrane after ethanol exposure which instigates severing of the cilia [34].

Since worms achieve forward motion via the interaction of their cilia and mucosal layer, one possible contributing factor to gross movement inhibition by EtOH may be the reduction of mucus production, as evidenced by differential H&E staining of EtOH-treated sections (Figure 6C). Very few published H&E stainings have been done in planarians [for example see 35], so the exact identification of cells in these sections is often not clear. However, we do know that hematoxylin is a basic stain that colors nuclei and ribosomes blue/purple, and eosin is an acidic stain that colors protein-rich cytoplasm and extracellular matrix pink/red. Extrapolating from drawings in historical planarian histology papers [36], [37], it is likely that the cells which are affected by EtOH treatment are mucosal cells called rhabdites.

Rhabdites are located in the planarian epithelium and swell upon contact with water to produce mucus [24], consistent with the white fluid-filled areas seen in control epithelia (Figure 6C1). Rhabdite-precursor cells are acidophilic and located beneath the epithelium [38], [39], consistent with the bright pink eosin staining we observed below the epithelium in controls (Figure 6C1, C3). Since planarians secrete a layer of mucus in which cilia can propel the body (ciliary gliding) without muscle involvement [15], [24], it is possible that loss of cilia and mucus production are connected, although this has not been tested.

Reduced mucus production may also contribute to the “scrunched” morphology of EtOH-treated worms. The mucosal layer provides protection from predators, toxins and microorganisms [40], [41]. Thus without an intact, uniform mucosal layer, worms are more vulnerable. One potential way to maintain a layer over the entire surface, despite decreased mucus production, would be to decrease surface area with a “scrunched” morphology. It is unclear what the mechanism for mucus loss might be. It could be that the ethanol interferes with the rhabdite's ability to properly swell on contact with water, thereby inhibiting mucus production. It is also possible that resources are preferentially used for regrowing cilia, rather than producing rhabdites, and so the rate of mucus production is decreased until the cilia are fully regrown. Further analyses will be required to determine whether or not rhabdites themselves are lost with EtOH-treatment or if mucus production alone is inhibited.

Fine movement inhibition with EtOH immobilization does not appear to arise from mucus/cilia loss, as it is the stationary muscle movements which are inhibited. The fine movements observed in treated worms consist of stretching and twisting the body, especially the head. Since planarians use their muscles to negotiate obstacles by changing the shape and direction of their body, reserving cilia for forward motion, planarian fine movements are most likely muscle-mediated, and not ciliary, movements. Previous studies have also hypothesized that a low percent EtOH exposure affects “musculature-mediated” movements [14].

We hypothesize that EtOH influences planarian muscular movements at a neurogenic level. EtOH has been shown to change the firing activity of mouse brains, and to decrease the excitability of neurons in the central nervous system and inhibit synaptic currents in mammals [42]. Planarians possess a both central nervous system and most of the neurotransmitters commonly found in mammals [43]. Therefore it is possible that EtOH affects planarian synaptic transmissions in a similar manner. That EtOH does not affect myogenic contractions themselves is supported by the persistence of fine movement responses to tactile stimuli even after immobilization. It could be argued that the lack of cilia in EtOH-treated worms contributes to the cessation of fine movements (despite their obvious ability for muscle contractions), as the absence of ciliary gliding prevents EtOH-immobilized worms from encountering stimuli. However, the loss of neuronally-regulated photophobic responses to light in EtOH-treated planarians suggests that neurogenic activity is being affected. The specific effects of EtOH on either neuronal or muscular function in planarians have not been examined and remain an area for future investigations. In conclusion, a brief 1 hour treatment with 3% ethanol is a simple, new method to inhibit the fine movements of planarians, one that is an improvement over current methods of immobilization.

## Materials and Methods

### Colony Care

The asexual clonal line CIW4 of *Schmidtea mediterranea* was used and maintained as described [16], [17]. Specifically, animals were maintained at 19–20°C in 1× Montjuïc salts (worm water). Unless otherwise stated, worms 4–6 mm in length were used. Worms were starved for at least 1 week prior to use in experiments.

### EtOH Immobilization and Amputations

Worms were exposed to 3% ethanol, using 200 proof EtOH (Pharmco-Aper) and worm water, for 1 hour (unless otherwise stated), followed by 3× washes in plain worm water. Treated worms (removed from EtOH) were used for all experiments and scoring, as worms still in EtOH are prone to twitching. Due to evaporation, fresh 3% EtOH must be made just prior to each treatment. Unless noted, amputations were performed as in [6] and scored at 14 days.

### DiBAC-CC2 Imaging

DiBAC_4_(3) (DiBAC; bis-[1,3-dibarbituric acid]-trimethine oxanol) (Invitrogen) was used as in [5]. DiBAC was used at 0.475 µM (from a 1.9 mM stock in DMSO) in worm water. CC2-DMPE (CC2, N-(6-cholor-7-hydroxycoumarin-3-carbonyl)-dimyristoylphosphatidyl ethanolamine) (Biotium) was used at 5 µM in worm water (from a 5 mM stock in DMSO). 24-hour regenerating trunk fragments were incubated in CC2 for 30 minutes, washed 3×, and then placed in DiBAC for at least 30 minutes; regenerates were imaged while in DiBAC. Images were captured at 460 nm (CC2) and 517 nm (DiBAC) wavelengths (Fig. 1A & Fig. 8). CC2 is a membrane-bound voltage sensitive dye often used as a FRET partner with DiBAC, which is membrane soluble and fluoresces brighter in depolarized cells.

### RNAi Knockdown

In vitro double-stranded (ds)RNA to PC2 was prepared from a PCR template using T7 and T3 polymerases (Promega) and injected, both as described [18]. The injection schedule was a total of three injections (one injection per day of three pulses) for 3 consecutive days. At 2 weeks after injection, worms were assayed for inhibition of gross movements prior to use in experiments.

### Behavioral Analysis

Gross movements (Fig. 2) were quantified using a custom-made computer vision system [19]. Worms were kept in the dark prior to and throughout 10 minute trials (data recorded at 5 hertz). Coordinates were analyzed in Microsoft Excel, and J-Specimen (Ebiotics) was used to generate heat maps from Excel data. The colors used on heat maps are proportional to how often a spot was visited: black indicates the area was never visited, blue represents the fewest and red the most visits. For Fig. 4, movements were scored (following removal from EtOH) for both exposure period (treatment length) and recovery using a dissecting microscope, with a MI-150 FiberLite illuminator (Dolan-Jenner) set at exactly 50% (light source). Worms were scored for gross movement by placing graph paper (6 mm squares) beneath the Petri dish and recording the number of squares traveled by each worm during 3 minutes. Fine movements were scored at the end of 5 minutes.

### Histological Staining and Immunohistochemistry

For histology: worms were fixed using Carnoy's Fixative as previously described [20], and then stored in EtOH at −20°C. Worms were embedded in paraffin blocks and 5 µm sections were cut with a Leica PM2255 microtome and mounted onto slides. For staining, sections were treated with xylene (Acros Organics) to remove paraffin and rehydrated through a series of alcohols and distilled water. Sections were stained with hematoxylin and eosin (Fisher) following standard protocols. Samples were dehydrated through a series of alcohols and xylene and mounted with the permanent mounting medium Permount (Fisher). For immunohistochemistry: worms were fixed using Carnoy's Fixative, and then stored in MeOH at −20°C. Worms were processed as in [21] using either an α-phosphorylated histone H3, 1∶250 (Upstate) and an HRP-conjugated anti-Rabbit with TSA-Alexa568 anti-HRP (Tyramide Signal Amplification Kit, Molecular Probes); or with anti-acetylated tubulin, 1∶1,000 (T6793, Sigma) and a goat anti-mouse Alexa 488, 1∶400 (Sigma).

### Imaging and Figure Preparation

Whole worm images, immunohistochemistry images, and the image in Fig. 6A2 were taken by a Nikon SMZ1500 microscope with a Retiga 2000R camera (Q-Imaging) using Q-Capture imaging software. Cilia (60×), timelapse (20×), and DiBAC/CC2 (4×) images were taken by an Olympus BX-61 microscope equipped with a Hamamatsu ORCA AG CCD camera using either IPLabs or MetaMorph software. Histological sections were examined on a Zeiss Axioskop 2 plus light microscope with 10× (Fig. 6B) and 40× (Fig. 6C) objectives, using an Axiocam HRC digital camera (captured at 2600 dpi). Adobe Photoshop was used to orient and scale images and improve image clarity. Data were neither added nor subtracted; original images available upon request.

### Statistical Analyses

Error bars for % phenotype (Fig. 5B) and % movement (Fig. 4C) were calculated using 95% Confidence Intervals (CI). For significance, Microsoft Excel was used to perform Student's t-Tests, assuming 2-tailed distribution of two independent samples with unequal variance.
